# Gm6PGDH1, a Cytosolic 6-Phosphogluconate Dehydrogenase, Enhanced Tolerance to Phosphate Starvation by Improving Root System Development and Modifying the Antioxidant System in Soybean

**DOI:** 10.3389/fpls.2021.704983

**Published:** 2021-08-13

**Authors:** Cheng Li, Kangning Li, Mingming Zheng, Xinyi Liu, Xianlong Ding, Junyi Gai, Shouping Yang

**Affiliations:** ^1^Key Laboratory of Biology and Genetic Improvement of Soybean (General, Ministry of Agriculture), Jiangsu Collaborative Innovation Center for Modern Crop Production, National Center for Soybean Improvement, Soybean Research Institute, Nanjing Agricultural University, Nanjing, China; ^2^State Key Laboratory of Crop Genetics and Germplasm Enhancement, Nanjing Agricultural University, Nanjing, China; ^3^Ministry of Agriculture (MOA) Key Laboratory of Plant Nutrition and Fertilization in Lower-Middle Reaches of the Yangtze River, Nanjing, China

**Keywords:** soybean (*Glycine* max), *Gm6PGDH1*, phosphate starvation, root system development, reactive oxygen species

## Abstract

Phosphorus plays an important role in plant growth and development, and is an important limiting factor for crop yield. Although previous studies have shown that 6-phosphogluconate dehydrogenase (6PGDH) plays an important role in plant resistance to adversity, its response to low phosphorus (P) stress remains unknown. In this study, we reported the cloning and characterization of a cytosolic 6PGDH gene, *Gm6PGDH1*, which enhanced the tolerance to phosphate (Pi) starvation by improving root system development and modifying the antioxidant system in transgenic plants. *Gm6PGDH1* was highly expressed in the root at full bloom stage, and strongly induced by Pi starvation. The results from intact soybean composite plant and soybean plant, both containing a *Gm6PGDH1*-overexpressing construct, showed that *Gm6PGDH1* was involved in root system development, and subsequently affected P uptake under Pi-deficient conditions. Meanwhile, the accumulation of reactive oxygen species (ROS) in the root tip of transgenic soybean was reduced, and the activity of ROS-scavenging enzymes was enhanced compared with those of the wild type under Pi-deficient conditions. Interestingly, we found that the overexpression of *Gm6PGDH1* weakened the response of several other important Pi-answer genes to Pi starvation, such as some purple acid phosphatases (PAPs) and redox-related genes. In addition, the results from a virus-induced gene silencing (VIGS) indicated that *Gm6PGDH1* might have functional redundancy in soybean, and the results from a heterogeneous transformation system showed that overexpressing *Gm6PGDH1* also enhanced tolerance to Pi starvation in transgenic *Arabidopsis*. Together, these results suggested the great potential of *Gm6PGDH1* in crop breeding for low Pi tolerance.

## Introduction

Phosphorus is one of the most important mineral elements that affect plant growth and metabolism, which plays an important role in the regulation of photosynthesis, respiration, and a series of enzymes (Jain et al., 2007). Plant roots obtain essential phosphorus (P) only by absorbing inorganic phosphate (Pi) of soil. Although the overall P content in soil is high, most P is converted to highly insoluble and tightly bound forms, resulting in a large amount of P immobilized in the soil and unusable by plants (Schachtman et al., 1998). Nearly half of the arable land in the world has insufficient availability of Pi, which limits crop productivity (Hinsinger, 2001). Using Pi fertilizer solely to ensure production will not only increase economic costs but also cause environmental pollution (Bennett et al., 2001). Furthermore, the non-renewable nature of Pi fertilizers means that cheap sources of P, such as Pi rocks, will be exhausted within the next 60–90 years (Hammond et al., 2004). Therefore, in the face of increasing P shortage and P pollution, it is undoubtedly one of the most correct and effective means to establish efficient P genotypes in crops (Cong et al., 2020).

To cope with Pi deficiency, plants have developed many adaptive mechanisms to enhance the availability and uptake of P from soil, including modification in root architecture by stimulating the growth of lateral roots and root hairs to maximize root surface area, increased exudation of small molecular organic acids and activities of purple acid phosphatases (PAPs), and symbiosis with mycorrhizal fungi (Veneklaas et al., 2012). Root is the only organ that plants absorb Pi, and its favorable development is the key to adapting to Pi starvation (Chevalier et al., 2003; Péret et al., 2011; Oldroyd and Leyser, 2020). So far, through the unremitting efforts of botanists, hundreds of genes regulating root development in a low-Pi environment have been discovered, which have made important contributions to the cultivation of low-Pi tolerant crops. For example, *OsPTF1*-overexpressing transgenic rice showed significantly higher total root length and root surface area under Pi-deficient conditions, which results in a higher instantaneous Pi uptake rate over their wild-type counterparts (Yi et al., 2005); overexpression of *OsMYB2P-1* in rice led to longer primary roots and adventitious roots than wild-type plants under Pi-deficient conditions, enhancing tolerance to Pi starvation (Dai et al., 2012); the knockout of *OsARF16* led to primary root (PR), lateral root (LR), and root hair losing sensitivity to low Pi response (Shen et al., 2013). These studies indicate that root system development is closely related to low Pi resistance of plants. However, the molecular mechanisms of root system development in response to Pi starvation remain unclear.

Reactive oxygen species (ROS) signal molecules mainly include hydrogen peroxide (H_2_O_2_), superoxide radicals (O^2–^), and hydroxide ions (OH^–^), which play an important role in maintaining the normal growth of plants and improving their stress resistance (Huang et al., 2019). ROS production induced by adverse environmental stimuli is a common phenomenon in plants (Saed-Moucheshi et al., 2014), including Pi starvation (Shin et al., 2005). Plants can respond to stress through ROS to adapt to the environment, but excessive ROS in plants will cause damage to seedlings, cell structure and function, enzyme system, and DNA (Schieber and Chandel, 2014; Waszczak et al., 2018). In plant cells, ROS can be scavenged through several major ROS-scavenging enzymes, such as superoxide dismutase (SOD), ascorbate peroxidase (APX), catalase (CAT), glutathione peroxidase (GPX), and peroxidase (POD), and these ROS-scavenging systems are crucial to plant tolerance under stresses (Foyer and Noctor, 2005; Dvorak et al., 2020). ROS has been shown to be involved in plant redox processes, and the redox state of plant cells is highly correlated with cellular ROS production and processing (Nadarajah, 2020). Studies have shown that the redox balance of root cells was important to sustain the activity of root apical meristem under Pi starvation (Gutierrezalanis et al., 2018). In recent years, many genes related to oxidative stress that control the level of ROS have been found by functional classification of Pi-response genes with the development of gene sequencing technology, suggesting that redox reactions in roots plays an important role in plant adaptation to low Pi (Wu et al., 2003; Misson et al., 2005; Zhang et al., 2019), but few redox-related genes have been functionally validated under low Pi stress conditions.

The oxidative pentose phosphoric pathway (OPPP) is a major metabolic pathway of glucose degradation. OPPP can produce a large amount of nicotinamide adenine dinucleotide phosphate (NADPH, reduced coenzyme II), which provides the main reducing power for biosynthesis, and the intermediate metabolites it produces also provide raw materials for many biosynthesis (And and Emes, 2000; Kruger and Schaewen, 2003). Both glucose-6-phosphate dehydrogenase (G6PDH) and 6-phosphogluconate dehydrogenase (6PGDH) are considered as rate-limiting enzymes in OPPP, and are widely distributed over the cytoplasm and plastids of higher plants (Ap Rees, 1985). For years, G6PDH has been reported to be associated with tolerance responses to abiotic stress in many plants, such as *Arabidopsis* (*Arabidopsis thaliana)*, rice (*Oryza sativa*), wheat (*Triticum aestivum*), and soybean (*Glycine* max) (Nemoto and Sasakuma, 2000; Liu et al., 2013; Zhang et al., 2013; Yang et al., 2019a). However, compared with G6PDH, there were few studies on the relationship between 6PGDH and plant tolerance response to abiotic stress. Current studies show that 6PGDH plays an important role in regulating biological metabolite resistance to damage to the external environment, and may improve the stress resistance of rice by enhancing the glucose metabolism pathway (Hou et al., 2007).

Soybean is an important grain and oil crop in the world, and the main source of human high-quality protein and feed protein for animal husbandry (Sample et al., 1980). It can interact with rhizobia to form a nodule for symbiotic nitrogen fixation, so Pi deficiency is more likely to be one of the important limiting factors for high yield of soybean (Ohlrogge, 1960). To date, few studies have linked 6PGDH to the response of plants to Pi starvation. In this study, we cloned *Gm6PGDH1*, a cytosolic 6PGDH gene from soybean, and found that it might enhance tolerance to Pi starvation by improving root system development and modifying the antioxidant system in soybean.

## Materials and Methods

### Bioinformatics Analysis of *Gm6PGDH1*

The NCBI database^1^ was used to search for genetic information. The SMART database^2^ was used to analyze amino acid sequences. The BioXM 2.6 software was used to predict the molecular weight and isoelectric point of the gene. ClustalX 1.83 was used for multiple alignments. Neighbor-joining phylogenetic trees were generated using the MEGA 5.1 program.

### Recombinant *Gm6PGDH1* Protein Expression and Enzyme Activity Assay

The expression vector pET29a-*Gm6PGDH1* was constructed using the Gateway Technology with a One Step Cloning Kit (Vazyme Biotech, Nanjing, China) according to the protocol of the manufacturer. The sequences of the primers are provided in Supplementary Table 1. The pET29a-*Gm6PGDH1* plasmid was transformed into the *Escherichia coli* Rosetta (DE3) strain, and the expression of *Gm6PGDH1* was induced with 0.5 mM IPTG to generate the putative recombinants. Then, the His-tagged Gm6PGDH1 proteins were extracted and purified under native conditions using Ni Focurose 6FF (IMAC), and the products were detected by 10% Sodium dodecyl sulfate-polyacrylamide gel electrophoresis (SDS-PAGE).

The measurement of *Gm6PGDH1* enzyme activity was based on a previous method, with some changes (Šindelář et al., 1999). The recombinants were ultrasonic, broken and centrifuged at 4°C, and the supernatant was enzyme crude extracts. The total reaction volume of the assay was 2 ml containing a 1.8-ml reaction buffer (50 mM Triethanolamine, pH 7.5, 5 mM MgCl_2_, 0.18 mM NADP^+^, 1 mM 6PG) and 0.2 ml enzyme crude extracts. Absorbance changes at 340 nm were monitored using a visible spectrophotometer (722N, Shanghai Precision Science Instrument, Shanghai, China). The absorbance value was observed and recorded once every 10 s, and the reaction time was 2 min. A total of three replicates were included in this experiment.

### Plant Materials and Growth Conditions

Soybean Williams 82 plants were used in this study. Soybean seeds normally were germinated and grown in a growth chamber under controlled photoperiod and temperature (16 h light; 25°C/8 h dark; 20°C). Five-day-old seedlings (removal of cotyledons) were transferred to a vermiculite medium containing a modified Hoagland solution for 11 days, with 1 mM KH_2_PO_4_ (1 mM Pi, Pi-sufficient) or 2.5 μM KH_2_PO_4_ (2.5 μM Pi, Pi-deficient) as the P supply. On the 10th day, the Pi-deficient group was re-supplied with 1 mM Pi for 1 day and was called R1d. The nutrient solution was refreshed every 3 days. Soybean tissues were collected at specific times. All the samples were stored at −80°C prior to RNA extraction.

### Quantitative Real-Time PCR

Three biological replicates, each comprising three individual plants, were used for quantitative real-time PCR (RT–qPCR). Total RNA was extracted using the RNA Prep Pure Plant Kit (TIANGEN, Beijing, China). RT–qPCR analysis was performed as described previously (Li et al., 2020). The relative level of expression was calculated using the formula 2^–△*Ct*^ or 2^–△△Ct^. Actin (GLYMA_08G146500) or Actin2-8 (AT3G18780) was used as an endogenous control to normalize the samples. All the primers used for RT-qPCR are listed in Supplementary Tables 2, 3.

### Subcellular Localization of *Gm6PGDH1*

The coding region of *Gm6PGDH1* was amplified with specific primers (Supplementary Table 1), and cloned into the pJIT166-GFP vector to construct the pJIT166-Gm6PGDH1-GFP vector. The fusion (pJIT166-Gm6PGDH1-GFP) and control (pJIT166-GFP) constructs were transformed into *Arabidopsis* mesophyll protoplast using the polyethylene glycol method (Yoo et al., 2007). The fluorescence of GFP was imaged with a Zeiss LSM780 confocal microscope (Carl Zeiss, SAS, Jena, Germany).

### Development of Transgenic *Arabidopsis*

The fused plasmid vector (pCAMBIA3301-Gm6PGDH1) was transformed into wild-type *Arabidopsis* (Col-0) plants using the *Agrobacterium*-mediated floral-dip procedure (Clough and Bent, 1998). Transgenic plants were selected on a Basta (20 mgL^–1^) medium, and the selected plants were identified by PCR and RT-qPCR (Supplementary Figures 6A,B). Homozygous T3 or T4 seeds and wild-type seeds, which were harvested in the same environment, were used for research.

### *Gm6PGDH1* Overexpression in Soybean Composite Plants

To confirm the function of *Gm6PGDH1* in soybean, the full-length *Gm6PGDH1* coding sequence was inserted after the CaMV35S promoter, resulting in pCAMBIA3301-Gm6PGDH1 constructs with a GFP label (the original GUS label was replaced by the GFP label). The sequences of the primers are provided in Supplementary Table 1. Then, the overexpression vector and the empty vector were separately introduced into *Agrobacterium rhizogenes* K599, which was used to infect soybean (Williams 82) seedlings in order to obtain composite soybean plants with transgenic hairy roots (Attila et al., 2007). When transgenic hairy roots grew approximately 8–10 cm long, a small part was harvested for RT-qPCR identification (Supplementary Figure 1). The primary roots of the transgenic composite plants were cut off, and plants were transferred to Hoagland vermiculite medium containing 1 mM Pi or 2.5 mM Pi for 14 days after 2 days of growth with Hoagland nutrient solution. Each transgenic root represented an independent transgenic line; for each Pi treatment, six independent transgenic lines were included. After 14 days of growth, leaves and hairy roots were harvested separately to determine dry weight and total P concentration. Several hairy roots were taken for further RT-qPCR analysis. An independent transgenic line originating from a composite soybean plant with a transgenic hairy root was considered as a semi-biological replicate. A total of three replicates were included in this experiment.

### Soybean Whole-Plant Transformation

The fused plasmid vector (pCAMBIA3301-Gm6PGDH1) was transformed into soybean (Williams 82) using the *Agrobacterium*-mediated transformation method (Li et al., 2017b). Positive transgenic plants were screened in a greenhouse by leaf herbicide painting and PCR-based amplification of genomic DNA. T3-generation plants of homozygous independent transgenic lines were used for research.

### BPMV-Mediated Gene Silencing

A 329-bp DNA fragment of the *Gm6PGDH1* gene was PCR-amplified using gene-specific primers (Supplementary Table 1) and cloned into the BPMV RNA2 silencing vector to construct BPMV-Gm6PGDH1 vectors (Zhang et al., 2010). As described earlier, DNA-based BPMV constructs were inoculated in V1 stage soybean seedlings by mechanical friction (Pflieger et al., 2015). The empty BPMV vector was used as a control. Two weeks post-virus inoculation, the leaf edge of some of the plants was wrinkled and deformed (Supplementary Figure 5A), indicating successful infection. Then, small leaves of the plants with infection symptoms were harvested to isolate the RNA for verification of gene silencing by RT-qPCR (Supplementary Figure 5B). The infected plants were transferred to vermiculite medium containing 2.5 μM Pi Hoagland nutrient solutions for 7 days. Each infected plant represented one independent gene silencing line. One independent infected plant was considered as one biological replication. A total of three replicates were included in this experiment.

### Measurement of Chlorophyll Content

Five-day-old soy bean plants were grown in a Hoagland medium with two Pi levels (1 mM KH_2_PO_4_ and 2.5 μM KH_2_PO_4_) for 30 days. The nutrient solution was refreshed every 3 days. The chlorophyll content of the second newly unfolded leaves at the top was assayed with SPAD-502Plus (Konicaminolta, Japan).

### Measurement of Total P and Soluble Pi Concentration

For the plant total P concentration assay, fresh plant samples were heated at 75°C until completely dry, and then ground into powder separately. Approximately 0.05 g of the plant dry samples were weighed and digested with H_2_SO_4_ and H_2_O_2_. After cooling, the digested samples were diluted to 100 ml with distilled water. Then, the concentration of P in the solution was determined with 710 ICP-OES (Agilent Technologies, Santa Clara, CA, United States). Each experiment was repeated three times.

For the soluble Pi concentration assay, Tissue Inorganic Phosphorus Content Detection Kit (Solarbio, Beijing, China) was used. Each sample consisted of five seedlings, which were ground into fine powder in liquid nitrogen, repeated three times.

### Analysis of Root System Development

For transgenic soybean root analysis, an Expression 21000XL (Epson, Nagano, Japan) scanner with a root analysis system WinRHIZO 2020 (Regent, Canada) was used. Transgenic *Arabidopsis* photography and root system analysis were performed as described previously (Li et al., 2020).

Trypan blue was used for the analysis of root activity. The root tips (2 cm) of soybean seedlings were dyed with 0.1% (W/V) trypan blue for 3 min in a boiling water bath, washed with distilled water for 10 min, and the observed under an OLYMPUS CX31 stereo microscope with a SPOT-RT digital camera (Olympus, Melville, NY, United States) attached to a computer.

### Analysis of ROS Homeostasis

For ROS accumulation analysis, staining with Diaminobenzidine (DAB) of stress-treated seedlings was performed following the reported protocol (Daudi and O’Brien, 2012). H_2_O_2_ content was determined according to the method described previously, with slight modifications (Yang et al., 2019b). For the soybean plants, 0.2 g of the root tips were taken as a sample, and for *Arabidopsis*, five seedlings were taken as a sample. After overnight reaction, the absorbance was read at 390 nm, and H_2_O_2_ content was determined using a standard curve. Each experiment was repeated three times.

For activity of ROS-scavenging enzymes analysis, soybean root tips (0.2 g) were ground with a 1.6 ml ice-cold 50 mM phosphate-buffered saline (PBS) (pH 7.8). The homogenate was centrifuged at 16,000 × g for 20 min at 4°C, and the supernatant was used for the assays. The activities of SOD, POD, and APX were determined according to established protocols (Luo et al., 2020). Nicotinamide adenine dinucleotide phosphate (NADPH) content was determined by following the method described previously, with slight modifications (Matsumura and Miyachi, 1980; Wei et al., 2019). Homogenate plant tissue samples in 2 ml 0.1 M NaOH solution under ice bath conditions were then centrifuged at 10,000 × g for 20 min at 25°C, and 50 μl of the supernatant of the sampling solution was added with 300 μl of a mixture (0.1 M NaOH, 40 mM EDTA, 4.2 mM MTT, 16.6 mM PMS), followed by 450 μl of 0.1 mM NaCl, and the solution was kept for 5 min at 37°C. Then, the solution was mixed with 50 μl G6P dehydrogenase (0.5 KU/ml) and incubated at 37°C for 40 min. The reaction was terminated with 200 μl of saturated NaCl and centrifuged at 10,000 × g for 10 min at 25°C. The supernatant was discarded, and the precipitate was dissolved with 1 ml 95% ethanol (v/v). After the precipitation was completely dissolved, OD value was measured at 570 nm. Five *Arabidopsis* seedlings were taken as a sample, and three biological replicates were set in each sample. The NADPH content was expressed as OD_570_/plant FW.

### Statistical Analyses

Statistical analyses were performed using Microsoft Excel 2010 and the SAS System for Windows V10. Significant differences were evaluated by two-tailed Student’s *t*-test or one-way ANOVA and Duncan’s test. All test differences at *P* < 0.05 were significant. All the error bars were SD value.

## Results

### Protein Structure, Prokaryotic Induction, and Enzyme Activity Determination of *Gm6PGDH1*

**FIGURE 1:**
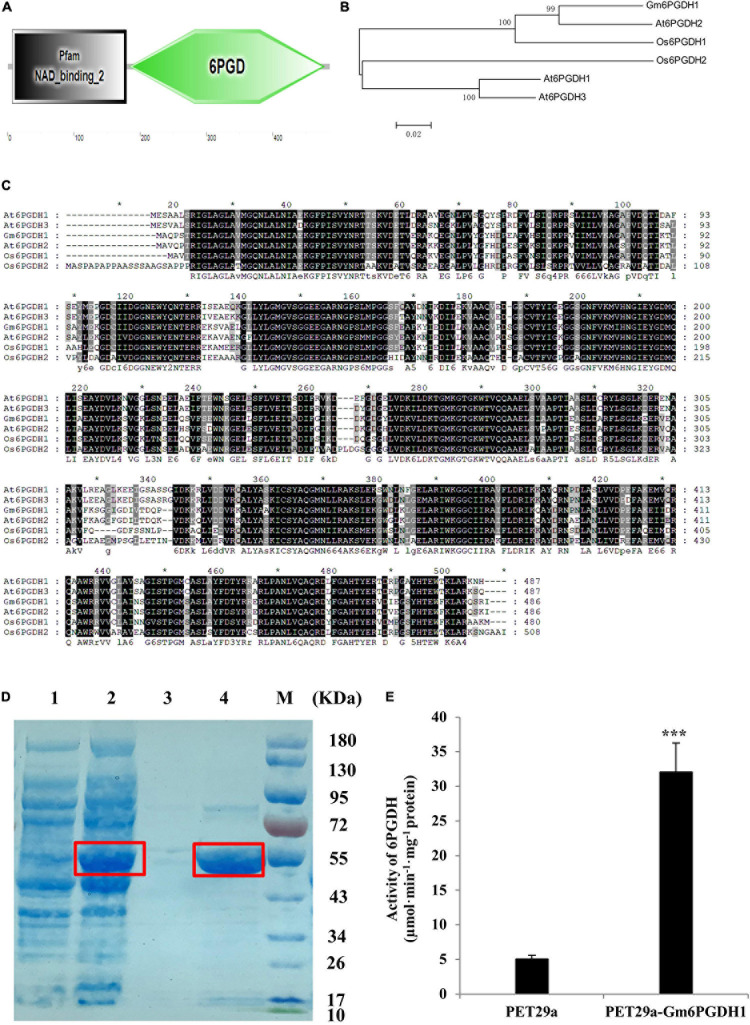
Protein structures, prokaryotic induction, and enzyme activity determination of *Gm6PGDH1*. **(A)** Domain analysis of Gm6PGDH1. **(B)** Phylogenetic tree constructed based on Gm6PGDH1 and the other plant 6PGDH proteins. **(C)** Protein sequence comparison between Gm6PGDH1 and *Arabidopsis* (At6PGDH1, At6PGDH2, and At6PGDH3) and rice (Os6PGDH1 and Os6PGDH2) 6PGDH proteins. Identical amino acids are shown with a black background, and analogous amino acids are shaded in gray. **(D)** Coomassie-stained 10% SDS-PAGE of cell extract. Lane 1: Rosetta (DE3) *Escherichia coli* strain expressing empty vector protein (PET29a); Lane 2: DE3 expressing His-tagged Gm6PGDH1 fusion protein (PET29a-Gm6PGDH1); Lane 3: Purified PET29aprotein; Lane 4: Purified PET29a-Gm6PGDH1 fusion protein; M: protein maker. The target protein was in the red frame. **(E)** GPDH enzyme activities from Gm6PGDH1 fusion protein. Data means ± SD (*n* = 3). Asterisks indicate a significant difference from the PET29a (Student’s *t*-test, ***P < 0.001).

A 6PGDH gene (GLYMA_08G254500) was obtained through BLAST searching against the soybean genome utilizing the reported *Arabidopsis* 6PGDH family protein gene *PGD2* as query (Hölscher et al., 2016), and it was named *Gm6PGDH1*. *Gm6PGDH1* was located on Gm8 (22503089-22506054) of soybean, and its full-length coding sequence (CDS) had 486 amino acids with a calculated molecular mass of 53.57 kD and an isoelectric point of 6.4. Amino acid sequence analysis showed that Gm6PGDH1 had a C-terminal 6GPD domain and an N-terminal NAD-binding domain (Figure 1A). Phylogenetic analysis was performed to determine the evolutionary relationship between Gm6PGDH1 and the other plant 6PGDH proteins, among which *Gm6PGDH1* was most closely related to At6PGDH2 (Figure 1B). The results of amino acid sequence alignment showed that Gm6PGDH1 was highly conserved, and that the homology between Gm6PGDH1 and At6PGDH1, At6PGDH2, At6PGDH3, Os6PGDH1, and Os6PGDH2 was 76.35, 90.12, 76.76, 85.65, and 70.95%, respectively (Figure 1C).

To further test whether Gm6PGDH1 exhibited an enzymatic activity, the prokaryotic expression vector of *Gm6PGDH1* was constructed. The recombinant protein generated by the *E. coli* Rosetta (DE3) strain expressing plasmid of Histagged Gm6PGDH1 was purified with a Ni-NTA column, and recombinant proteins produced by DE3 strain were detected by SDS-PAGE. A *Gm6PGDH1*-His fusion protein with an expected size of 54.39 kDa (consisting of target gene and histidine marker) was identified after Coomassie brilliant blue staining (Figure 1D). Meanwhile, the recombinant Gm6PGDH1 protein was assayed for its kinetic properties relative to the substrate NADP^+^. The recombinant proteins were ultrasonic-broken and centrifuged at 4°C, and the supernatant was taken as enzyme crude extracts. Then, the reaction buffer was added into the crude enzyme extracts of *Gm6PGDH1*-His, and the crude enzyme extracts of an empty carrier were used as the control. The relative activity of bacterial protein was calculated by the slope of the curve, and the results showed that the relative enzyme activity of the prokaryotic-induced *Gm6PGDH1* fusion protein was significantly higher than that of the empty carrier (Figure 1E).

### Expression and Subcellular Localization of *Gm6PGDH1*

**FIGURE 2:**
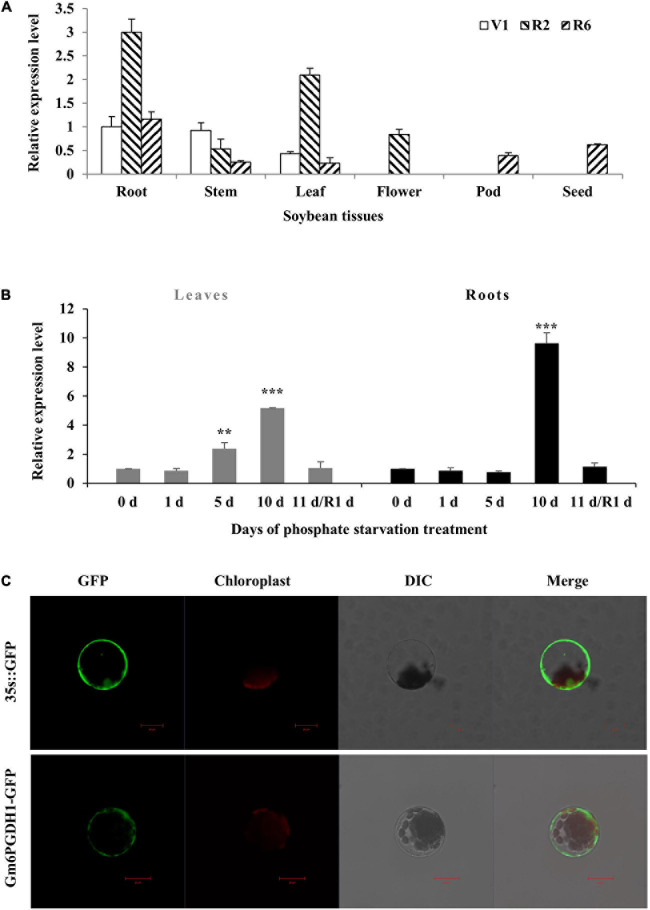
Expression patterns and subcellular localization of *Gm6PGDH1*. **(A)** Tissue-specific transcript abundance of *Gm6PGDH1* in root, stem, leaf, flower, pod, and seed at different developmental stages, the first trifoliate stage (V1), full bloom stage (R2), and full seed stage (R6). **(B)** Time course of the *Gm6PGDH1* expression in response to Pi starvation. R1d means resume Pi supply for 1 day. Actin was used as an internal control. Data means ± SD (*n* = 3). Data that are significantly different from the corresponding controls are indicated (Student’s *t*-test, **P < 0.01; ***P < 0.001). **(C)** Subcellular localization of the Gm6PGDH1 protein in protoplasts of *Arabidopsis*. Scale bar = 20 μm.

To investigate the transcript levels of *Gm6PGDH1* in specific tissues, the total RNA was extracted from root, stem, leaf, flower, pod, or seed of the soybean plants at first trifoliate (V1), full bloom (R2), and full seed (R6) stages. The RT-qPCR analysis showed that *Gm6PGDH1* was expressed in all the tissues examined, with the highest expression in root at the R2 stage and lowest in leaf at the R6 stage (Figure 2A). The expression patterns of *Gm6PGDH1* in the root was altered significantly that the largest transcription was observed at the R2 stage. A similar expression pattern was observed in the leaf (Figure 2A). Then, RNA samples from the root and leaf were used to evaluate the expression patterns of *Gm6PGDH1* to Pi starvation by RT-qPCR. The results showed that the expression levels of *Gm6PGDH1* significantly increased over time under low Pi conditions. After 10 days of treatment with Pi starvation, the expression level of *Gm6PGDH1* in the root and leaf both rose to the maximum value, but the expression level of *Gm6PGDH1* in the root was higher than that in the leaf (Figure 2B). Furthermore, the relative levels of *Gm6PGDH1* in both the root and the leaf dropped rapidly after recovery of Pi for a day (Figure 2B). These results suggested that *Gm6PGDH1* was involved in transcriptional response during soybean Pi starvation adaptions.

Previous literature suggested that there were two forms of 6PGDH in plants: cytosolic 6PGDH and plastidic 6PGDH, and that the N-terminal of plastid 6PGDH amino acid sequence had one more signal peptide sequence compared with cytosolic 6PGDH (Tanksley and Kuehn, 1985; Spielbauer et al., 2013). The domain analysis of Gm6PGDH1 showed no signal peptide at the N-terminal (Figure 1A), so it may be cytosolic 6PGDH. To verify the subcellular localization of Gm6PGDH1, a Gm6PGDH1-green fluorescent protein (GFP) fusion construct and a control construct containing only GFP were generated. Both constructs were transformed into the mesophyll protoplasts of *Arabidopsis* and observed under a confocal laser scanning microscope. The free GFP alone was present throughout the whole cell, whereas Gm6PGDH1-GFP was specifically located in the cytosol, which demonstrated that Gm6PGDH1 was cytosolic 6PGDH (Figure 2C).

### Functional Analysis of *Gm6PGDH1* in Soybean Composite Plants

**FIGURE 3:**
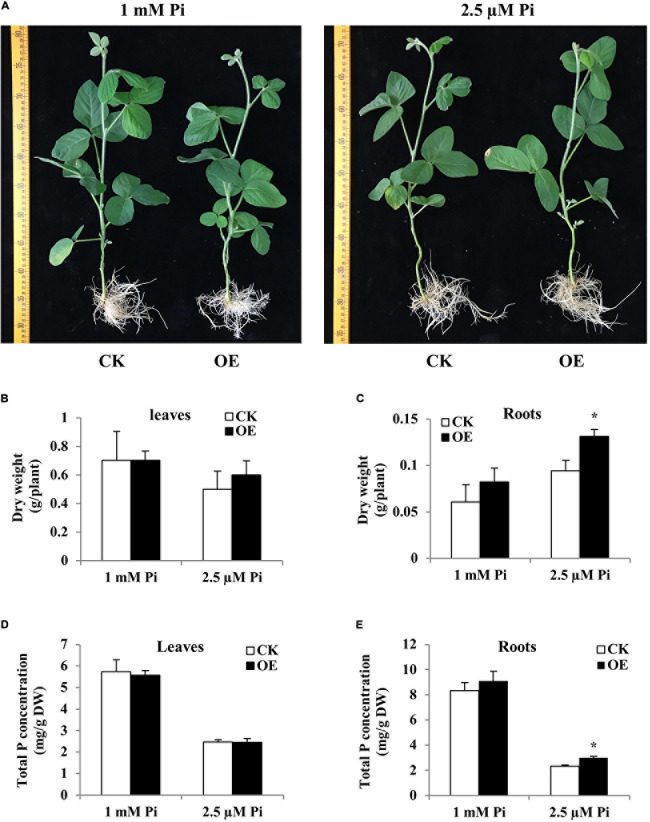
Comparison of control (CK) and *Gm6PGDH1*-overexpressing composite soybean plants under two Pi level conditions. Composite soybean plants with transgenic hairy roots were grown with a normal nutrient solution for 2 days, and then the plants were switched to a nutrient solution containing 1 mM or 2.5 μM KH_2_PO_4_. After 14 more days, the leaves and roots were separately harvested for analysis. **(A)** Phenotype of composite soybean plants. Scale unit: cm. **(B)** Dry weight of leaves. **(C)** Dry weight of roots. **(D)** Total P concentration of the leaves. **(E)** Total P concentration of the roots. CK represents the composite plants transformed with the empty vector; OE indicates *Gm6PGDH1*-overexpressing composite plants. DW means dry weight. Data are means ± SD of three biological replicates. Asterisks indicate significant differences between OE and CK (Student’s *t*-test, *P < 0.05).

Composite plants with transgenic hairy root attached to wild-type (WT) shoot can be obtained by hairy root transformation, which provides a fast and effective way for functional analysis of genes expressed in soybean root (Zhang et al., 2014; Xue et al., 2017). To produce composite soybean plants consisting of a wild-type shoot with transgenic roots, *Agrobacterium rhizogenes* should be injected into the cotyledonary node and/or the proximal part of the hypocotyl, which would lead to the development of hairy roots at the infection site. In this case, the chimeric plants have a strong root connection based on direct outgrowth from the vasculature (Attila et al., 2007). By RT-qPCR analysis, the expression level of *Gm6PGDH1* in transgenic hairy roots was about twofold over empty vector control hairy roots (Supplementary Figure 1). Under Pi-deficient conditions, the control plants showed more obvious symptoms of P deficiency than the *Gm6PGDH1*-overexprssing composite plants, such as fewer buds and deep color leaves (Figure 3A). Meanwhile, the dry weight and total P concentration of the roots of the composite transgenic plants were significantly higher than those of the control plants under Pi-deficient conditions, while there was no significant difference under Pi-sufficient conditions (Figures 3C,E). These results suggested that the expression of *Gm6PGDH1* in soybean root may affect the P efficiency of plant root by regulating root system development under Pi-deficient conditions. In this experiment, the overexpression of *Gm6PGDH1* did not lead to changes in dry weight and total P concentration of the composite transgenic plants leaf, regardless under Pi-deficient or Pi-sufficient condition (Figures 3B,D), that part of the reason may be the shorter processing time.

As an important category of genes involved in the Pi starvation rescue system, PAPs play an important role in plant Pi acquisition (Raghothama, 1999; Tomscha et al., 2004; Liang et al., 2010). Previous studies have shown that the transcript level of 66% GmPAP genes were induced or increased by Pi-deprivation treatment in soybean (Li et al., 2012). In order to further understand the regulatory role of *Gm6PGDH1* in Pi starvation, the transcription of five GmPAP genes was analyzed in hairy roots of transgenic composite plants. In the hairy roots of the control plants, Pi starvation significantly induced the expression of the five GmPAP genes (Supplementary Figure 2), which was similar to previous reports (Li et al., 2012, 2017a; Kong et al., 2018). In composite plant hairy roots with overexpressing *Gm6PGDH1*, however, no significant induction of *GmPAP14* and *GmPAP21* expression was detected (Supplementary Figure 2). In addition, the induction ratio of the *GmPAP3*, *GmPAP15*, and *GmPAP17* transcripts in the composite plants hairy roots overexpressing *Gm6PGDH1* was reduced by 50–80% compared with the control plants under Pi-deficient conditions (Supplementary Figure 2). There was no significant difference in the expression levels of these genes between composite plants hairy roots with overexpressing *Gm6PGDH1* and the control under Pi-sufficient conditions (Supplementary Figure 2). These results suggested that the overexpression of *Gm6PGDH1* attenuates the response to Pi starvation of several Pi-starvation inducible GmPAP genes in hairy roots of transgenic composite plants.

### Overexpression of *Gm6PGDH1* Enhanced Tolerance to Pi Starvation by Improving Root System Development of Soybean Under Pi-Deficient Conditions

**FIGURE 4:**
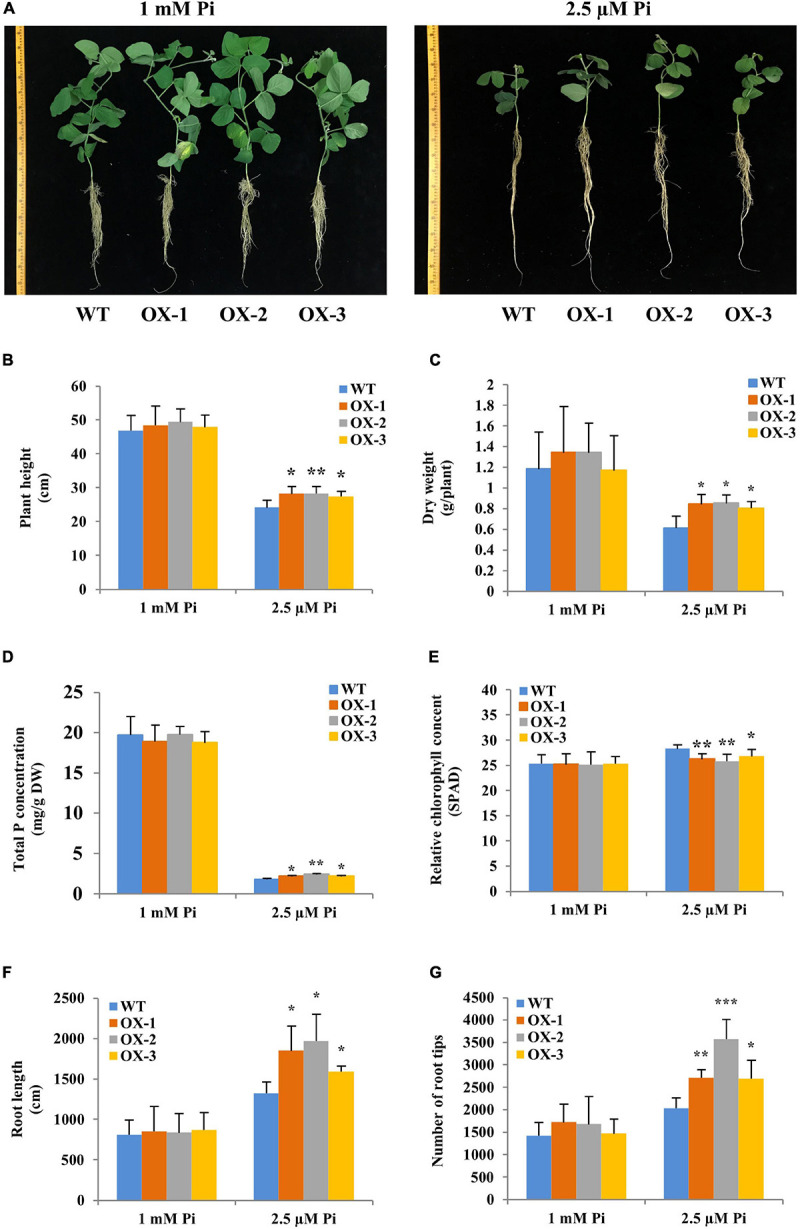
Overexpression of *Gm6PGDH1* enhances tolerance to Pi starvation in transgenic soybean. Five-day-old seedlings (removal of cotyledons) were exposed to nutrient solutions containing two levels of Pi, 1 mM Pi (Pi-sufficient), or 2.5 μM Pi (Pi-deficient) for 30 days. **(A)** Phenotypes of wild type (WT) and *Gm6PGDH1*-overexpressing lines (OX-1, OX-2, and OX-3) in response to two levels of Pi treatment. Scale unit: cm. **(B–G)** Plant height, fresh weight, total P concentrations, chlorophyll content, the total root length, and the number of root tips were recorded at the end of the treatment period. Data are means of three replicates with SD. Asterisks show that the values are significantly different between the transgenic lines and the WT at the same time point (Student’s *t*-test, *P < 0.05, **P < 0.01, ***P < 0.001).

Then, we generated three stable overexpressing soybean lines (OX-1, OX-2, and OX-3) to better investigate the role of *Gm6PGDH1* response and adaptation to Pi starvation (Supplementary Figures 3A,B). The T3 progeny of 5-day-old transgenic soybean lines and WT seedlings was exposed to a nutrient solution containing a high level of Pi (1 mM Pi, Pi sufficient) and a low level of Pi (2.5 μM Pi, Pi deficient) for 30 days (removal of cotyledons). The three *Gm6PGDH1*-overexpressing lines showed no significant growth difference compared with WT when grown with the Pi-sufficient solution (Figure 4). However, the *Gm6PGDH1*-overexpressing lines showed better growth than the WT plants when grown with the Pi-deficient solution (Figure 4A). There was a significant difference in plant height and dry weight between WT and *Gm6PGDH1*-overexpressing plants under Pi-deficient condition (Figures 4B,C). At the same time, the total P concentrations of the *Gm6PGDH1*-overexpressing lines were higher than in the WT under Pi-deficient conditions (Figure 4D). Since Pi starvation will lead to a relative increase in chlorophyll content (Devaiah et al., 2009), we monitored the chlorophyll content of all the plants and found that the chlorophyll content of transgenic plants under Pi-deficient conditions was significantly lower than that of WT (Figure 4E). These results showed that the overexpression of *Gm6PGDH1* increased the resistance of soybean to Pi starvation.

Since Pi starvation could cause changes in plant root architecture (Péret et al., 2011), we further observed the root development of transgenic soybean under Pi starvation. Under Pi-sufficient conditions, no significant differences in root architecture were observed between the WT and *Gm6PGDH1*-overexpressing lines (Figures 4F,G). However, the total root length in the *Gm6PGDH1*-overexpressing lines was significantly longer than that in WT under Pi-deficient conditions (Figure 4F). The number of root tips in *Gm6PGDH1*-overexpressing lines was significantly higher than that in WT under Pi-deficient conditions (Figure 4G). In addition, trypan blue can enter dead cells and appear blue (Zhang et al., 2011), so we tried to use it to explore the difference in root activity between transgenic soybean lines and WT. Under Pi-deficient conditions, the staining degree of soybean root tip (more accurately the meristematic zone) increased compared with that under P-sufficient conditions, indicating that the number of dead cells in the root tip increased (Supplementary Figure 4). However, we found that the staining degree of transgenic lines was lighter than that of WT, indicating higher root activity of *Gm6PGDH1*-overexpressing lines than WT under Pi-deficient conditions (Supplementary Figure 4). These results suggested that the improved growth of the *Gm6PGDH1*-overexpressing lines under Pi-deficient conditions may be partly due to better root system development.

### Overexpression of *Gm6PGDH1* Had a Positive Effect on the Antioxidant System of Soybean Under Pi-Deficient Conditions

**FIGURE 5:**
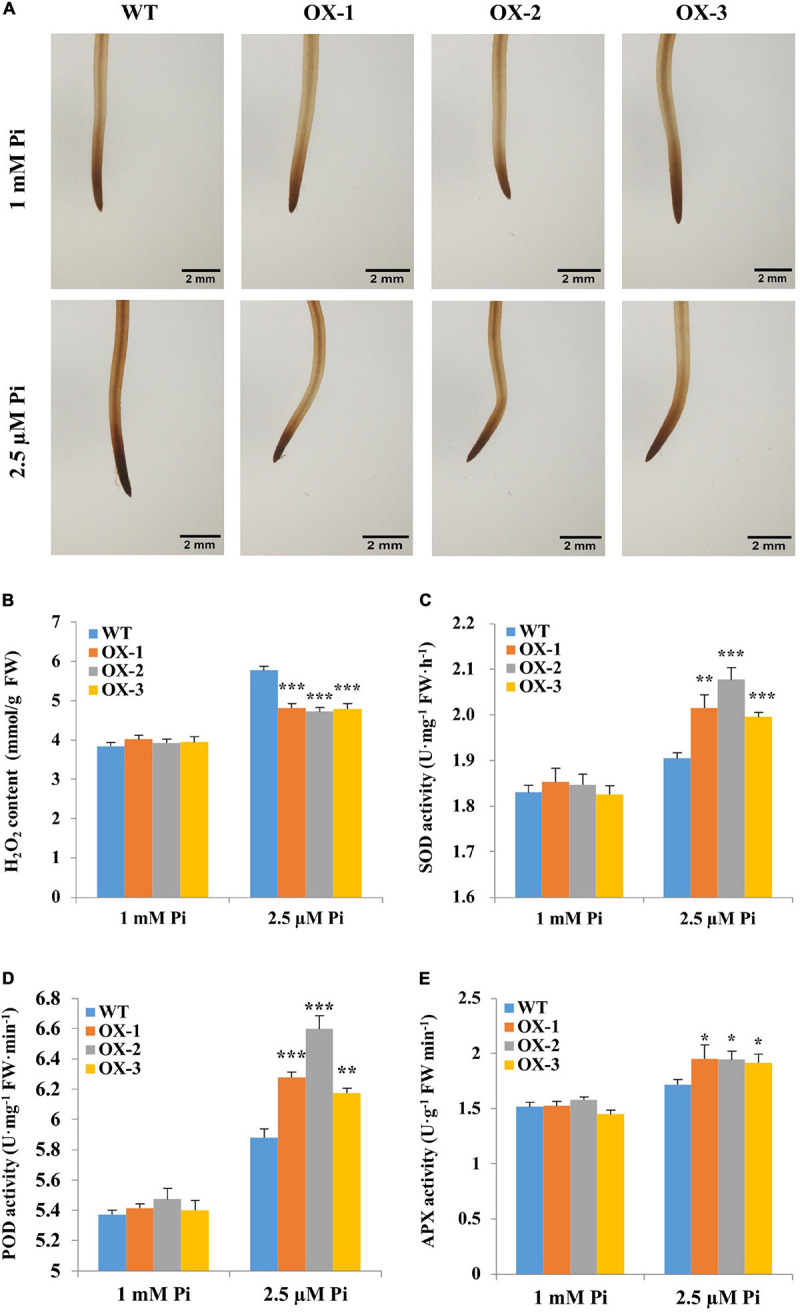
Changes in levels of H_2_O_2_ accumulation and activities of ROS-scavenging enzymes of soybean root tips during Pi starvation. Five-day-old soybean seedlings (removal of cotyledons) were exposed to nutrient solutions containing two levels of Pi, 1 mM Pi (Pi-sufficient) or 2.5 μM Pi (Pi-deficient) for 10 days. **(A)** Results from DAB staining to detect H_2_O_2_. Scale bar = 2 mm. **(B)** H_2_O_2_content. **(C)** Superoxide dismutase (SOD) activity. **(D)** Peroxidase (POD) activity. **(E)** Ascorbate peroxidase (APX) activity. The root tips of wild-type (WT) and *Gm6PGDH1*-overexpressing lines (OX-1, OX-2, and OX-3) are used as substrates for detection. These data were measured on day 10 of both Pi treatments. Data are means of the three replicates with SD. Asterisks show that the values are significantly different between the transgenic lines and the WT at the same time point (Student’s *t*-test, *P < 0.05, **P < 0.01, ***P < 0.001).

As the OPPP is widely involved in plant redox balance, and 6PGDH is its rate-limiting enzyme (Balzergue et al., 2017), we naturally thought that the overexpression of *Gm6PGDH1* might affect the redox system of transgenic soybeans (Lehmann et al., 2009). The T3 progeny of 5-day-old *Gm6PGDH1*-overexpressing transgenic soybean and WT seedlings were exposed to a nutrient solution containing a high level of Pi (1 mM Pi, Pi sufficient) or a low level of Pi (2.5 μM Pi, Pi deficient) for 10 days (removal of cotyledons). As ROS is a major factor causing oxidative stress, we examined the accumulation of one major ROS, H_2_O_2_. The root tips of all the treated soybean materials were dyed with DAB. As shown in Figure 5A, Pi starvation accentuates the staining of upper root in the expansion and elongation zones, while the *Gm6PGDH1*-overexpressing lines were stained to a lighter extent compared with that of WT under Pi-deficient conditions, implying that less ROS was produced in the transgenic soybean lines. Quantitative measurements further demonstrated that H_2_O_2_ contents in the root tips of the *Gm6PGDH1*-overexpressing lines were remarkably lower than that of WT (Figure 5B). These results indicated that transgenic lines had better oxidative stress tolerance in a low Pi environment. The important role of ROS-scavenging enzymes in ROS homeostasis prompted us to assess the enzyme activities of SOD, POD, and APX in the root tips of soybean (Gill and Tuteja, 2010). After 10 days of Pi starvation, it was noticeable that the three enzyme activities of the *Gm6PGDH1*-overexpressing lines were significantly higher than those in the WT (Figures 5C–E). No similar difference was found under Pi-sufficient conditions.

**FIGURE 6:**
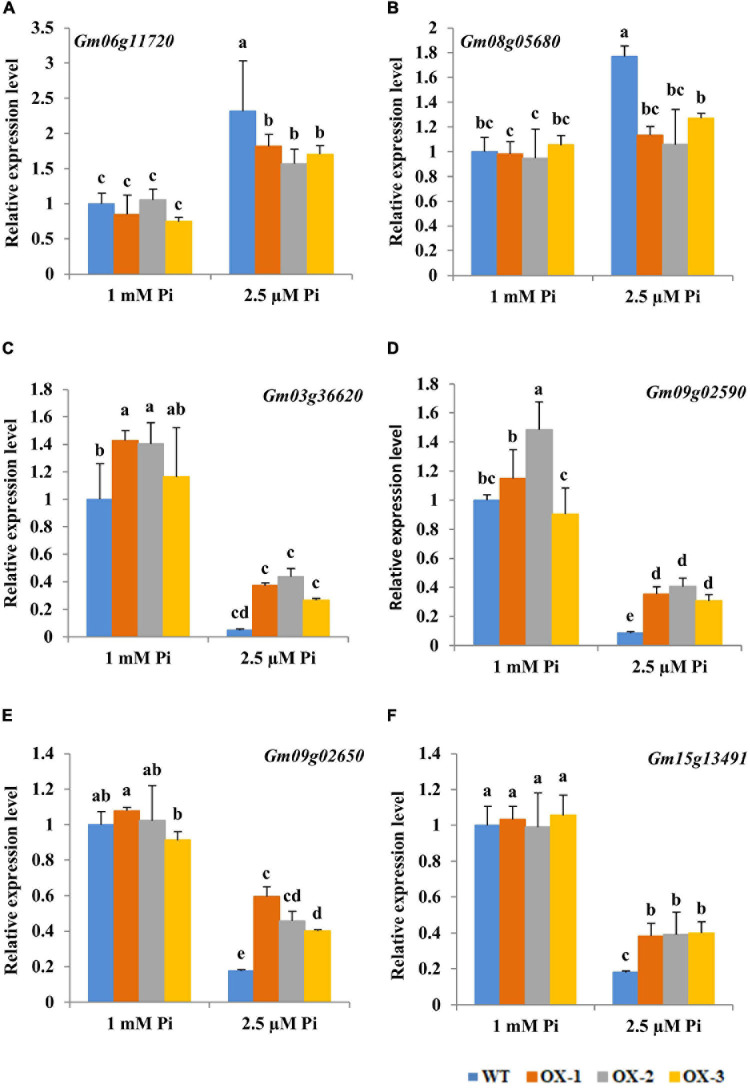
Changes in transcript levels for genes involved in the antioxidant system of soybean root tips during Pi starvation. Five-day-old soybean seedlings (removal of cotyledons) were exposed to nutrient solutions containing two levels of Pi, 1 mM Pi (Pi-sufficient) or 2.5 μM Pi (Pi-deficient) for 10 days. **(A)** *Gm06g11720*, **(B)** *Gm08g05680*, **(C)** *Gm03g36620*, **(D)** *Gm09g02590*, **(E)** *Gm09g02650*, **(F)** *Gm15g13491*. Actin was used as an internal control. Data are means of the three replicates with SD. Means with different letters are significantly different (one-way ANOVA, Duncan, *P* < 0.05).

To further elucidate the mechanisms underlying the regulation of antioxidant system by *Gm6PGDH1*, RT-qPCR was performed to monitor the expression of six oxidative stress-related genes identified by previous transcriptome to be associated with Pi starvation (Zeng et al., 2016). Under Pi-sufficient conditions, the expression levels of these genes showed no significant difference between *Gm6PGDH1*-overexpressing lines and WT, except that the expression levels of *Gm03g36620* and *Gm09g02590* were increased in some transgenic soybean lines compared with the WT (Figures 6A–F). At the same time, under Pi-deficient conditions, the transcription level of *Gm06g11720* and *Gm08g05680* increased (Figures 6A,B), while the transcription level of *Gm03g36620*, *Gm09g02590*, *Gm09g02650*, and *Gm15g13491* decreased (Figures 6C–F), which was similar to the results of previous studies (Zeng et al., 2016). Interestingly, the expression of these genes in response to Pi starvation was attenuated in transgenic lines (Figures 6A–F). For example, the expression of *Gm06g11720* under Pi-deficient conditions was 2.32 times than that under Pi-sufficient conditions in WT, while the expression of *Gm06g11720* under Pi-deficient conditions was 2.14, 1.49, and 2.28 times more than that under Pi-sufficient conditions in OX-1, OX-2, and OE-3, respectively (Figure 6A).

### Effect of Silencing *Gm6PGDH1* in Soybean Response to Pi Starvation

To further characterize the role of *Gm6PGDH1* in soybean response to Pi starvation, we attempted to knock down *Gm6PGDH1* by virus-induced gene silencing (VIGS). VIGS mediated by Beanpod mottle virus (BPMV) has been successfully performed to study the function of soybean genes involved in defense and other processes, such as *GmMPK4s*, *GmWRKY58*, and *GmWRKY76* (Liu et al., 2011; Yang et al., 2016). Two weeks post virus inoculation, some plants showed fold deformation of leaf margin (Supplementary Figure 5A), and RT–qPCR analysis showed that the plants inoculated with BPMV-Gm6PGDH1 vectors displayed a reduction of approximately 60% in the transcript levels of *Gm6PGDH1* when compared with control plants (Supplementary Figure 5B). The identified plants were subjected to Pi starvation for 7 days (Supplementary Figure 5C). There was no significant difference in the dry weight and total P concentration of the leaves and roots between BPMV-Gm6PGDH1-silenced plants and control plants (Supplementary Figures 5D,E). These results showed that the VIGS of *Gm6PGDH1* did not affect dry weight and total P concentration of soybean leaves and roots under low Pi conditions.

### *Gm6PGDH1* Improved Root System Development and Pi Accumulation, and Attenuated Oxidative Damage in Transgenic *Arabidopsis* Under Pi-Deficient Conditions

**FIGURE 7:**
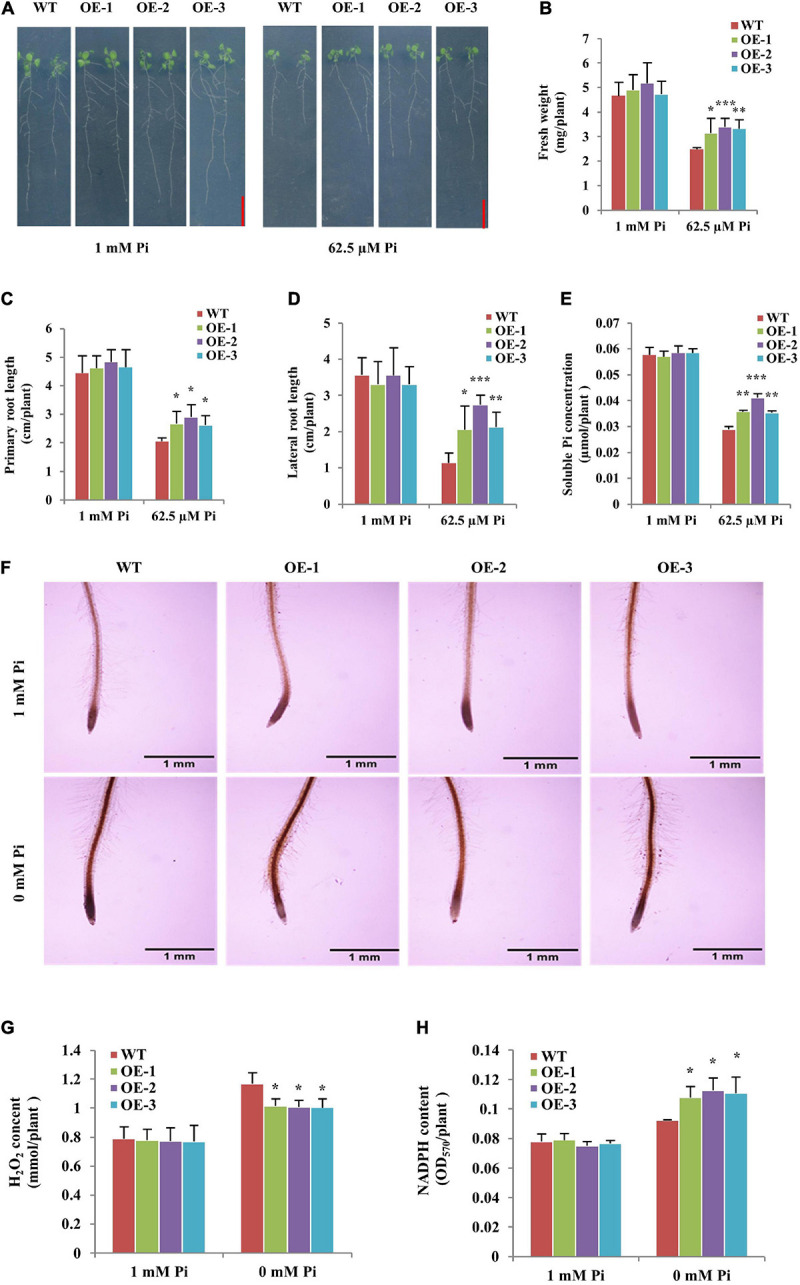
Overexpression of *Gm6PGDH1* improved root system development and Pi accumulation, and affected the oxidative damage in *Arabidopsis* under low Pi conditions. **(A)** Wild-type (WT) and *Gm6PGDH1*-overexpressing transgenic *Arabidopsis* (OE-1, OE-2, and OE-3) were grown in a Hoagland medium with 1 mM Pi or 62.5 μM Pi on vertically oriented Petri dishes for 14 days. Bars = 1 cm. **(B–E)** The fresh weight, primary root length, total lateral root length and soluble Pi concentration were determined in WT and transgenic *Arabidopsis* plants treated as above. Three independent experiments were performed. **(F)** Photographs showing DAB staining of 7-day-old WT and transgenic *Arabidopsis* seedlings treated with a medium with 1 mM Pi and 0 mM Pi for 36 h, respectively. Bars = 1 mm. H_2_O_2_ levels **(G)** and enzyme activity of nicotinamide adenine dinucleotide phosphate (NADPH) **(H)** in all lines treated as **(F)**. Data are means ± SD (*n* = 3). Data significantly different from the corresponding controls are indicated (Student’s *t*-test, *P < 0.05; **P < 0.01; ***P < 0.001).

In order to further verify the role of *Gm6PGDH1* in plant resistance to Pi starvation, *Gm6PGDH1*-overexpressing *Arabidopsis* plants were constructed. Seeds of T3 transgenic *Arabidopsis* and the WT were vertically cultured in a plant gel medium with high Pi (1 mM Pi) or low Pi (62.5 μM Pi) for 14 days. Under high Pi conditions, there was no significant difference in the growth status between the transgenic *Arabidopsis* lines and WT (Figures 7A,B). However, under low Pi conditions, the transgenic *Arabidopsis* lines grew much better than WT (Figure 7A). The fresh weight of the transgenic *Arabidopsis* lines was greater than that of WT under low Pi conditions (Figure 7B). At the same time, we found that the short-term low Pi supply significantly inhibited the growth of *Arabidopsis*, especially the root growth (Figure 7A), indicating that the length of primary and lateral roots length of plants grown with low Pi was shorter (Figures 7A,C,D). However, the primary and lateral roots of the transgenic *Arabidopsis* lines were significantly longer than those of the WT after 14 days of treatment with low Pi (Figures 7C,D). Furthermore, the soluble Pi concentration of the transgenic plants was significantly higher than that of WT under low Pi conditions (Figure 7E). These results indicated that the overexpression of *Gm6PGDH1* significantly promoted root system development and Pi accumulation in transgenic *Arabidopsis* under low Pi conditions.

Then, the ROS levels in the *Gm6PGDH1*-overexpressing *Arabidopsis* lines and WT were explored. We treated 7-day-old WT and transgenic *Arabidopsis* lines in an agar medium with high Pi (1 mM Pi) and low Pi (0 mM Pi) for 36h, then used DAB to stain the root tips of all the plants. Under low Pi conditions, the color of plant root was heavier, reflecting the elevated level of ROS (Figure 7F). However, the slighter coloration of DAB staining was observed in transgenic *Arabidopsis* lines root tip compared with WT under low Pi conditions (Figure 7F). Meanwhile, quantitative measurements showed that the content of H_2_O_2_ in the transgenic *Arabidopsis* lines was markedly lower than that in the WT under low Pi conditions (Figure 7G). Furthermore, we measured the NADPH content of the plants, since the OPPP contributes to the production of NADPH to support antioxidant enzyme activity (Valderrama et al., 2006; Lehmann et al., 2009). We found that the content of NADPH in the transgenic *Arabidopsis* lines was higher than that in WT under low Pi conditions (Figure 7H). These results suggested that the overexpression of *Gm6PGDH1* might alleviate the root tip ROS accumulation by improving the content of NADPH under low Pi conditions.

## Discussion

The plant OPPP has been shown to be related to plant growth and development, and to have responded to a variety of environmental stresses (Šindelář et al., 1999; Nemoto and Sasakuma, 2000; Spielbauer et al., 2013; Long et al., 2016). However, as one of the two key enzymes of the OPPP, compared with G6PDH, botanists pay less attention to 6PGDH. In this study, we cloned a cytosolic 6PGDH gene from soybean and named it *Gm6PGDH1*. Prokaryotic induction and enzyme activity determination of Gm6PGDH1 indicated that it was a protein with 6PGDH activity, and that it was like the previously reported 6PGDH (Redinbaugh and Campbell, 1998). The RT-qPCR results showed that *Gm6PGDH1* was widely expressed in various tissues of soybean, and that the expression level was highest in roots at the R2 stage, which was different from the expression pattern of *Os6PGDH1* in rice. The *Os6PGDH1* expression was high in inflorescence, low in the root and embryos, and almost absent in the leaf (Huang et al., 2003). Furthermore, the *Gm6PGDH1* expression in the root and leaf at the R2 stage has higher levels than at other stages, which may be related to the increased dependence of soybean on OPPP. The reason is that soybean grows vigorous during this period, and OPPP can promote cell division and biosynthesis (Wu et al., 2015). It is worth noting that the phylogenetic analysis and amino acid sequence alignment indicate that Gm6PGDH1 was most closely related to At6PGDH2 (Figures 1B,C); however, they might not be functionally similar. Previous studies have shown that *At6PGDH2* was related to fertility (Hölscher et al., 2016), and the conclusion of this research was that *Gm6PGDH1* was involved in Pi starvation tolerance. Therefore, we speculated that 6PGDH of the different plants might have different expression modes and functions, even if they were relatively conservative.

Previous studies have shown that the transcription level of *Os6PGDH1* was induced by abiotic stresses such as high salt, low temperature, drought, and ABA (Hou et al., 2007), and we have found that *Gm6PGDH1* was strongly induced by Pi starvation. These results suggested that 6PGDH might be widely involved in plant resistance to abiotic stress, but we still did not know exactly how 6PGDH works. In this study, the overall data clearly demonstrated that *Gm6PGDH1* enhanced the tolerance of soybean to Pi starvation by increasing the root length and the number of root tips, as well as enhancing the activity of the ROS-scavenging enzymes to reduce ROS (H_2_O_2_) accumulation. A recent study showed that a low-Pi tolerant genotype (NN94156) soybean had a larger root system at low Pi levels compared with a low-Pi sensitive genotype (Bogao) soybean, and transcriptome analysis results suggested that NN94156 had more oxidation-reduction processes than Bogao under low Pi conditions (Zhang et al., 2020), which was similar to the results of this study. However, interestingly, the expression of several other important redox-related genes appeared to be inhibited in the root tip of the overexpressed *Gm6PGDH1* soybean lines under low Pi conditions. This seemed to contradict the data that the root tips of *Gm6PGDH1-*overexpressing transgenic soybean lines had a higher activity of ROS-scavenging enzymes and less ROS level compared with those of WT. The possible explanation was that the positive effect of *Gm6PGDH1* on root development and the antioxidant system promoted Pi uptake, thus improving the Pi balance in the root system, and leading to interference in the response of some redox-related genes to Pi starvation, which was like performance of the low phosphorus insensitive mutants (Sanchezcalderon et al., 2006). In addition, in the hair root of the transgenic soybean composite plants, some Pi-starvation inducible GmPAP genes were also inhibited in response to low Pi, which further proved that the expression change in *Gm6PGDH1* would produce crosstalk to the other Pi-answer genes.

It is generally believed that low Pi will change root structure, leading to suppressed primary roots and enhanced lateral root development (Péret et al., 2011). However, in this study, the overexpression of *Gm6PGDH1* had a positive effect on the development of both primary and lateral roots in transgenic *Arabidopsis* under low Pi conditions, suggesting that the regulation of *Gm6PGDH1* in the root system might be independent under low Pi conditions. Further studies have shown that the accumulation of ROS (H_2_O_2_) at the root tip of transgenic soybeans and *Arabidopsis* decreased compared with WT under low Pi conditions, so we speculated that the positive effect of *Gm6PGDH1* on plant root might be related to the ROS homeostatic state of the root tip (Tsukagoshi et al., 2010). Low Pi has been demonstrated to promote ROS accumulation, which stiffens the cell wall and represses root growth (Balzergue et al., 2017). Recent evidence demonstrated that H_2_O_2_, low pH, malate, iron accumulation, and blue light are all indispensable for primary root response under Pi starvation conditions in *Arabidopsis* (Zheng et al., 2019). This study showed that the overexpression of the Gm6PGDH1 gene in *Arabidopsis* decreases the H_2_O_2_ content in the root tip, and this may explain why the transgenic lines show longer primary roots compare with WT under phosphate starvation conditions according to the former results. In plant cells, ROS can be scavenged through several major ROS-scavenging enzymes, such as SOD, POD, and APX (Foyer and Noctor, 2005), and NADPH is also a key component in regulating the balance of ROS in cells (Ying, 2008; Jiao et al., 2013; Yang et al., 2019b). The results of enzyme activity determination showed that the activities of SOD, POD, and APX were enhanced in the root tip of transgenic soybeans compared with WT, and that NADPH content was enhanced in transgenic *Arabidopsis* compared with WT under low Pi conditions. In addition, the *Gm6PGDH1*-overexpressing transgenic soybean lines have a higher root activity than WT under low Pi conditions, which further supports the opinion of the authors.

It is worth noting that The VIGS of *Gm6PGDH1* had no effect on dry weight and total P concentration of the plants under low Pi conditions, suggesting that function redundancy might be present for *Gm6PGDH1* in soybean. In previous studies, double mutants of the maizecytosolic isozymes, PGD1 and PGD2, had no obvious phenotype, and the roots of the mutants still had 32% of the catalytic capacity of 6PGDH of the wild-type plants (Bailey-Serres et al., 1992; Averill et al., 1998), so we guess that, partly, soybean plastidic 6PGDH may complement some functions of *Gm6PGDH1*, but this needs further verification in the later stage. In addition, it is worth investigating whether any other genes in this family were involved in regulating phosphorus deficiency in plants. In addition, results from a heterogeneous transformation system showed that overexpression of *Gm6PGDH1* enhanced tolerance to Pi starvation in transgenic *Arabidopsis* by improving root system development and reducing ROS accumulation, which proved that the positive effect of *Gm6PGDH1* for plant to Pi starvation was not limited to soybean. Therefore, it will be interesting for a study to use *Gm6PGDH1* to enhance the Pi starvation resistance of other plants and to explore whether the 6PGDH of other plants has a similar function (Ding et al., 2020).

## Conclusion

In conclusion, we report the cloning and functional characterization of a cytosolic 6PGDH gene, *Gm6PGDH1*, and show that this gene may play an important role in the internal regulation of root development and antioxidant system under low Pi stress conditions. Meanwhile, this study provides new evidence for the role of ROS homeostasis balance in root system development under low Pi environment conditions, and a new idea for the development of soybean materials with low Pi tolerance. In addition, due to differences in soil environment and laboratory conditions, further studies to see if the advantage conferred by the altered expression of *Gm6PGDH1* is maintained in soil-based systems are being carried out in the laboratory.

## Data Availability Statement

The original contributions presented in the study are included in the article/Supplementary Material, further inquiries can be directed to the corresponding author/s.

## Author Contributions

CL and SY designed the experiments and drafted and edited the manuscript. CL, KL, MZ, XL, and XD performed the experiments. CL processed the data and analyzed the corresponding results. JG reviewed the manuscript. All authors approved the final version of the study.

## Conflict of Interest

The authors declare that the research was conducted in the absence of any commercial or financial relationships that could be construed as a potential conflict of interest.

## Publisher’s Note

All claims expressed in this article are solely those of the authors and do not necessarily represent those of their affiliated organizations, or those of the publisher, the editors and the reviewers. Any product that may be evaluated in this article, or claim that may be made by its manufacturer, is not guaranteed or endorsed by the publisher.

## Footnotes

1
https://www.ncbi.nlm.nih.gov/

2
http://smart.embl-heidelberg.de/

## Supplementary Material

Supplementary Figure 1 Expression levels of *Gm6PGDH1* in hairy roots of soybean composite plants.

Supplementary Figure 2 Expression levels of several GmPAP genes in *Gm6PGDH1*-overexpressing composite soybean plants.

Supplementary Figure 3 The *Gm6PGDH1*-overexpressing transgenic soybean was identified by PCR and quantitative real-time PCR (RT-qPCR).

Supplementary Figure 4 Root activity was detected by trypan blue staining.

Supplementary Figure 5 Silencing *Gm6PGDH1* had no effect on tolerance to phosphate starvation in soybean.

Supplementary Figure 6 The *Gm6PGDH1*-overexpressing transgenic *Arabidopsis* was identified by PCR and RT-qPCR.

Supplementary Table 1 Primer sequences were used for vector construction and detection.

Supplementary Table 2 Primer sequences for qRT-PCR analysis in soybean.

Supplementary Table 3 Primer sequences for qRT-PCR analysis in *Arabidopsis*.
