# Supplementary material for: Gm6PGDH1, a Cytosolic 6-Phosphogluconate Dehydrogenase, Enhanced Tolerance to Phosphate Starvation by Improving Root System Development and Modifying the Antioxidant System in Soybean
Source: Front Plant Sci. 2021 Aug 13;12:704983. doi: 10.3389/fpls.2021.704983 (PMC8414836; doi:10.3389/fpls.2021.704983)
Supplement: Supplementary Figure 1 — Expression levels of Gm6PGDH1 in hairy roots of soybean composite plants. [file Data_Sheet_1.pdf]

Figure S1

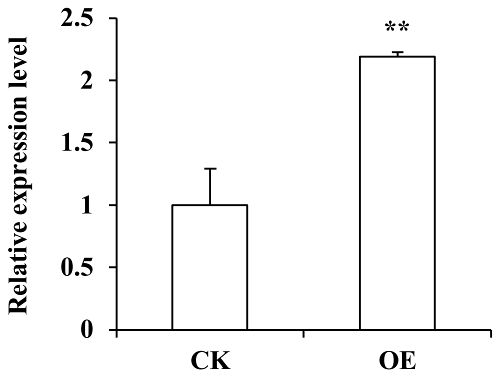

Figure S1. Expression levels of *Gm6PGDH1* in hairy root of soybean composite plants. CK represents soybean hairy root transformed with the empty vector; OE means transgenic soybean hairy root with overexpressing *Gm6PGDH1*. Values are the means of three independent samples, and bars indicate SD. Asterisk indicated a significant difference between OE and CK (Student's *t*-test, $**P < 0.01$).

Figure S2

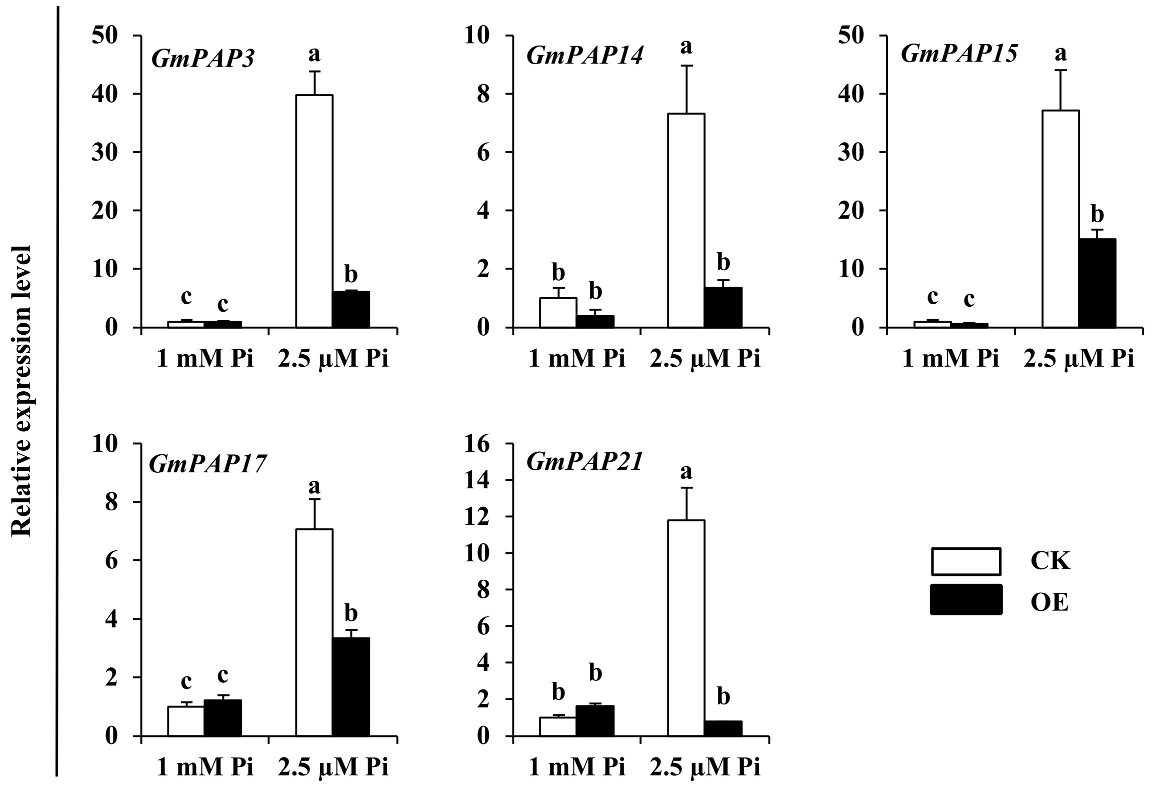

Figure S2. Transcript levels of some GmPAPs genes in *Gm6PGDH1*-overexpressing composite soybean plants. Plants were grown in nutrient solutions containing 1 mM or 2.5 μM KH_2PO_4 for 14 d, and transcripts in hairy roots were determined by RT-qPCR. CK represents soybean hairy roots transformed with the empty vector; OE indicates transgenic soybean hairy roots with overexpressing *Gm6PGDH1*. Values are the means of three independent samples, and bars indicate SD. Means with different letters are significantly different (one-way ANOVA, Duncan, $P < 0.05$).

Figure S3.

(a)

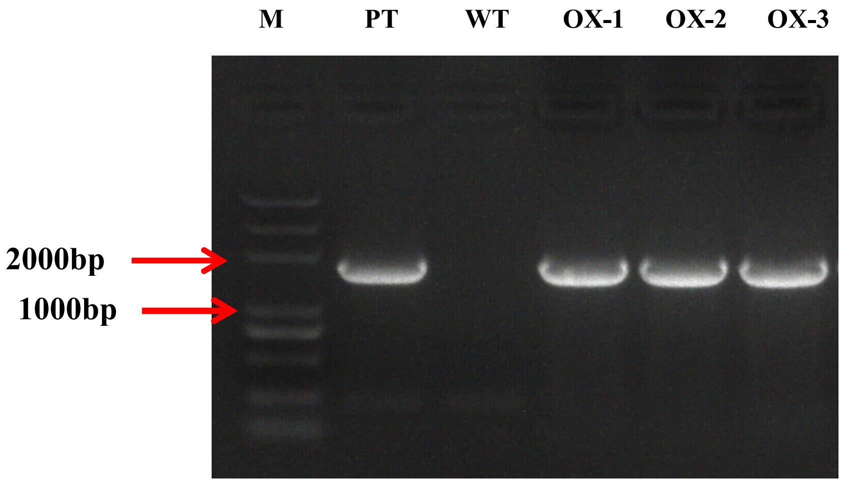

(b)

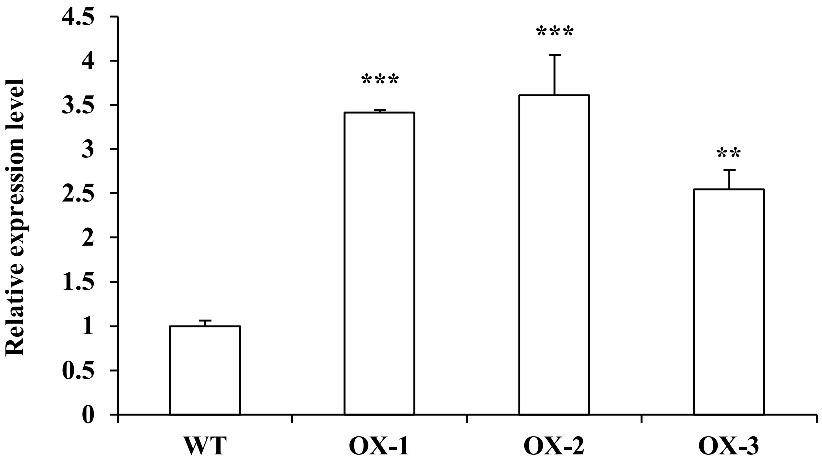

Figure S3. The *Gm6PGDH1*-overexpressing transgenic soybeans were identified by PCR (a) and RT-qPCR (b). (a) *Gm6PGDH1* gene has a size of 1461bp fragment**.** Lanes: M, DNA Marker DL5000; PT, the binary vector pCAMBIA3301-*Gm6PGDH1*; WT, no transformed wild type; OX-1, OX-2 and OX-3, three independent *Gm6PGDH1*-overexpressing transgenic soybean lines. (b) Expression levels of *Gm6PGDH1* in the WT and three transgenic soybean lines. Values are the means of three independent samples, and bars indicate SD. Asterisk indicated a significant difference between WT and each transgenic line (Student's t-test, $**P < 0.01$, $***P < 0.001$).

Figure S4.

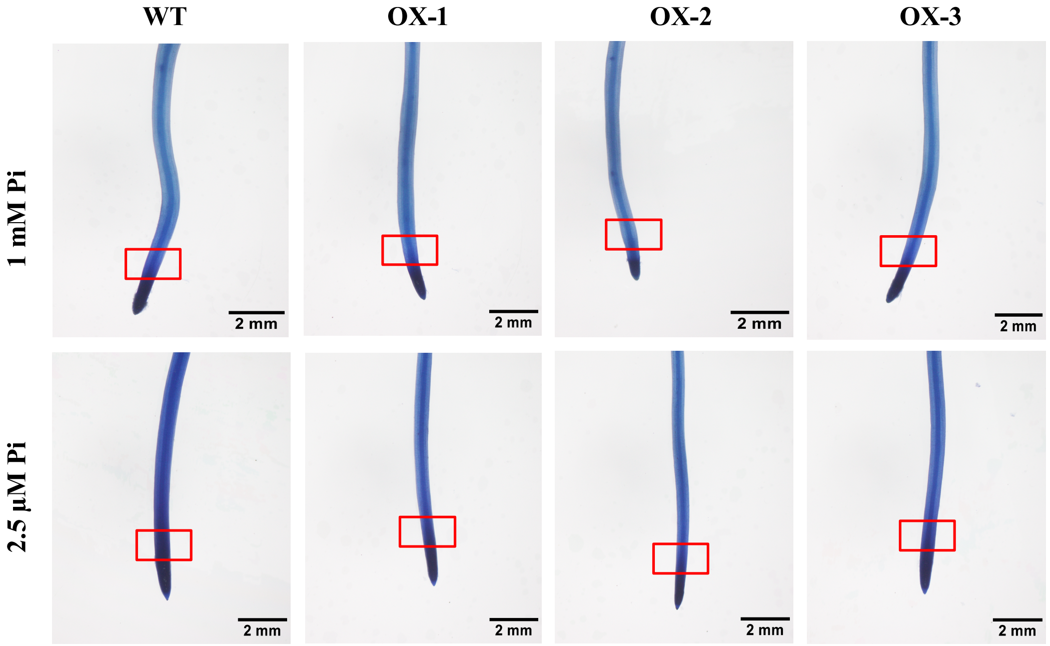

Figure S4. The root activity was detected by Trypan Blue staining. T3 progeny of 5 days old *Gm6PGDH1*-overexpressing transgenic soybeans (OX-1 to 3) and wild type (WT) seedlings were exposed to nutrient solution containing a high level of Pi (1 mM Pi, Pi-sufficient) and a low level of Pi (2.5 µM Pi, Pi-deficient) for 10 d (removal of cotyledons), then part of the root tips were taken for staining. The red box indicates the meristematic zone. Bars = 2 mm.

Figure S5.

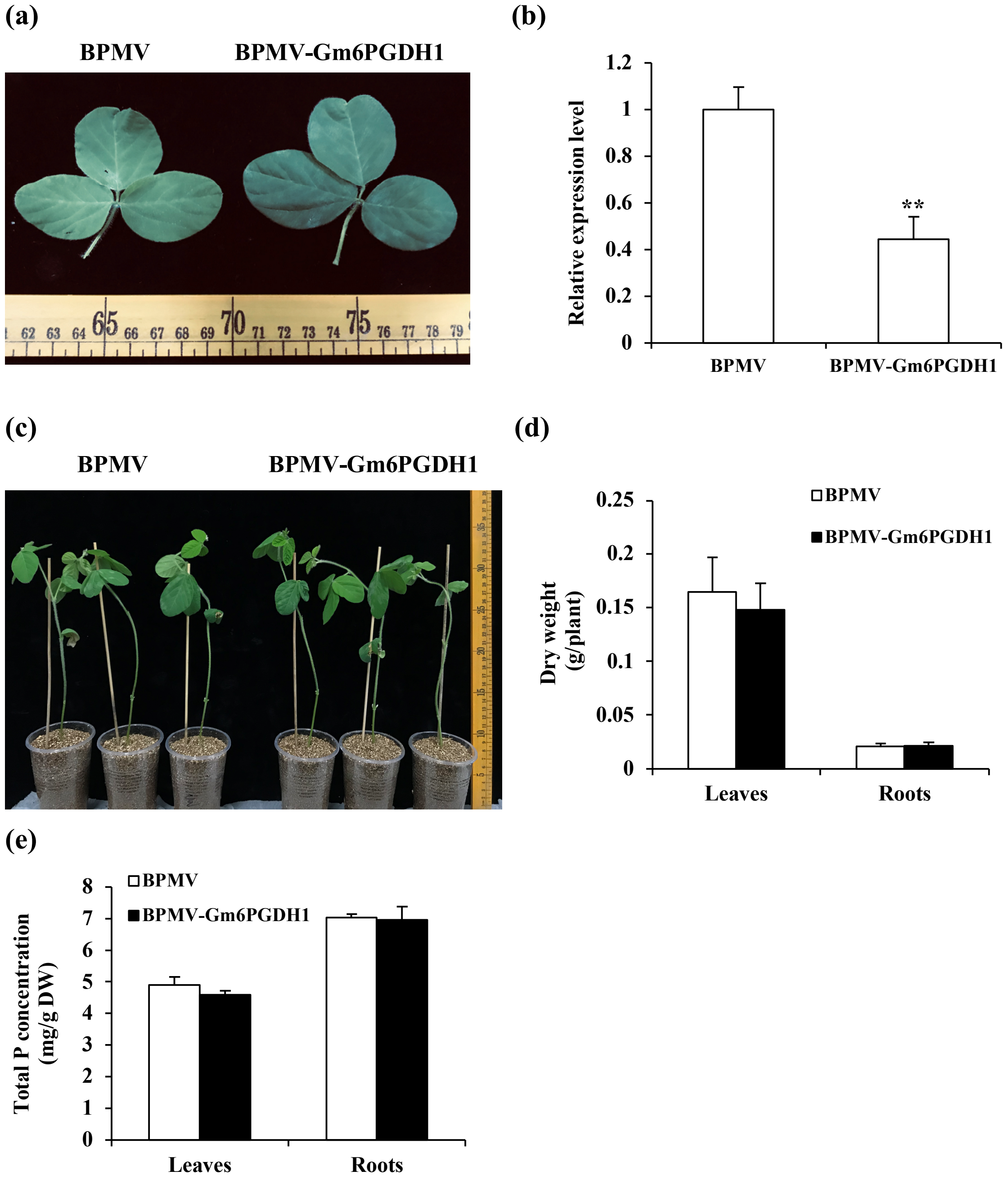

Figure S5. Silencing *Gm6PGDH1* had no effect tolerance to Pi starvation in soybean. (a) The picture of trifoliate leaves inoculated with BPMV (empty vector) and BPMV-*Gm6PGDH1* (silencing vector) were taken two weeks after inoculation. (b) Expression of *Gm6PGDH1* was performed by RT-qPCR using a soybean actin gene as control. (c) The picture of representative plants that were identified were taken seven days after Pi starvation. Dry weight (d) and total P concentration (e) of plants treated as above. The results shown are from three individual plants inoculated with empty vector (BPMV) or silencing vector (BPMV-*Gm6PGDH1*). Data means ±SD (n=3). Data significantly different from the corresponding controls are indicated (***P* < 0.01, Student's *t*-test). Scale unit in (a) and (c): cm.

Figure S6.

(a)

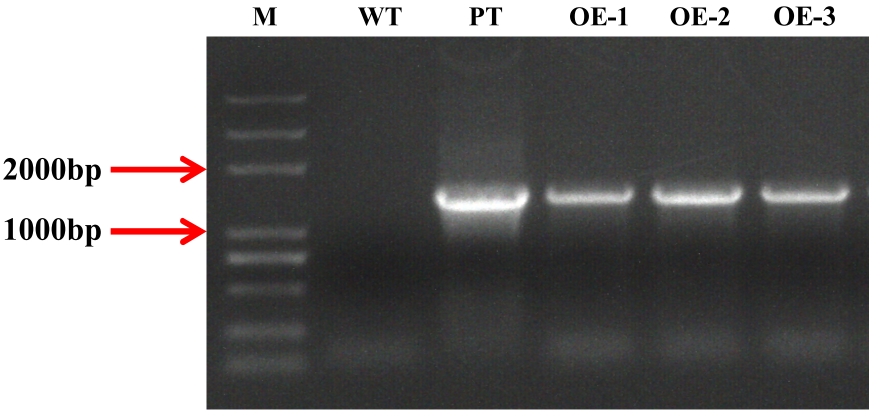

(b)

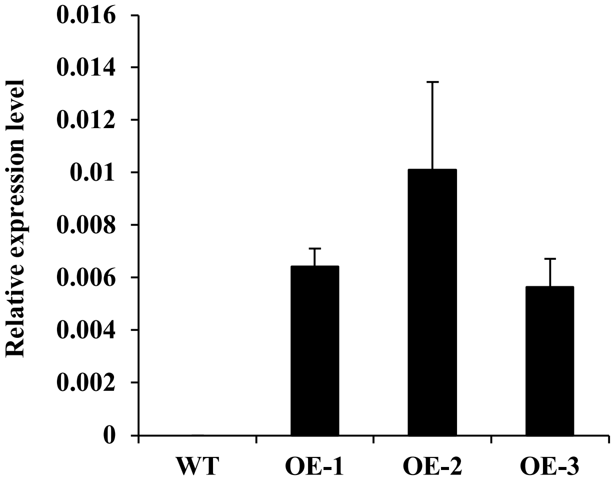

Figure S6. The *Gm6PGDH1*-overexpressing transgenic *Arabidopsis* was identified by PCR (a) and RT-qPCR (b). (a) *Gm6PGDH1* gene has a size of 1461bp fragment**.** Lanes**:** M, DNA Marker DL 5000; WT, wild type *Arabidopsis*; PT, the binary vector pCAMBIA3301-*Gm6PGDH1*; OE-1, OE-2 and OE-3, three independent *Gm6PGDH1*-overexpressing transgenic *Arabidopsis* lines. (b) Expression levels of *Gm6PGDH1* in the three transgenic *Arabidopsis* lines (OE-1 to 3). Each bar is the meaning of three replicates with SD.

Table S1. Primer sequences were used for vector construction and detection.

Name	Forward primer （5'-3'）	Reverse primer （5'-3'）
PET29a-*GmG6PDH1*	GCTGATATCGGATCCGAATTCATGGCTCAACCCTCAACAAGAATAG	GTGGTGGTGGTGGTGCTCGAGAATTCTAGACTGTTTGGCAAGCTTG
pJIT166-*GmG6PDH1*	GGCATGCATGGCTCAACCCTCAACAAGA	CCTCGAGAATTCTAGACTGTTTGGCAAGCTT
OE-*GmG6PDH1*	AACACGGGGGACTCTTGACAATGGCTCAACCCTCAACAAGAA	GCCCTTGCTCACCATAGATCTAATTCTAGACTGTTTGGCAAGC
pCAMBIA3301-GFP	AACACGGGGGACTCTTGACA	GCCCTTGCTCACCATAGATCT
VIGS	GATCCCACTTCCAAGGTTGATGAGACTGTA	TCGAGGCATCAAAGAGGGACCATTACG

Table S2. The primer sequences for qRT-PCR analysis in soybean.

Gene	Forward primer （5’-3’）	Reverse primer （5’-3’）
GmG6PDH1	TGGCTCAACCCTCAACAAGA	CAACCTTGGAAGTGGTTCGG
GmPAP3	CCAACAAGATGCTGCTAACGA	CAAAGAGGCCGAGTGAGATG
GmPAP14	TCAAGCAGCCCCTTCATTAG	AGTTTTCCTTCGGCAATCTTC
GmPAP15	TTCGGCAATGCCATCC	TTCGCAGTTGAAAATGCTCT
GmPAP17	GGTTTTAAGTGGCAGTTTGG	GCAACAATGGAGCTTTCTGA
GmPAP21	GCTGATGGTGTTTGGATTG	TGTTGGGTGTCAAAGTTGAG
Gm06g11720	TCTCTGGCACCGATTCACTT	AGGAGCTCCACTCCTAAACG
Gm08g05680	TCAGAGAGGAGCTCAGGACT	CGGCGACCTTAAGCATTTCA
Gm03g36620	CACTGCAGAGAAGGACTCCA	TCCCTAGCTGCCAATGCTAA
Gm09g02590	ATTTGGACCTGAGCACACCT	GTATCAGCACCAGGAGTGGA
Gm09g02650	CACTGGGAAGAAGGGATGGT	TGAGCACCTGAAAGTGCAAC
Gm15g13491	CTGCCTTTGCTGTTCAAGGT	AGAGTTGGACCAGGGTTTCC
Actin	CCTCAACCCAAAGGTCAACAG	GACCAGCGAGATCCAAACGAA

Table S3. The primer sequences for qRT-PCR analysis in *Arabidopsis*.

Gene	Forward primer （5’-3’）	Reverse primer （5’-3’）
GmG6PDH1	TGGCTCAACCCTCAACAAGA	CAACCTTGGAAGTGGTTCGG
Actin2-8	ACGGTAACATTGTGCTCAGTGGTG	CTTGGAGATCCACATCTGCTGGA
